# Reliable Single Chip Genotyping with Semi-Parametric Log-Concave Mixtures

**DOI:** 10.1371/journal.pone.0046267

**Published:** 2012-10-16

**Authors:** Ralph C. A. Rippe, Jacqueline J. Meulman, Paul H. C. Eilers

**Affiliations:** 1 Clinical Epidemiology, Leiden University Medical Center, Leiden, The Netherlands; 2 Institute of Mathematics, Leiden University, Leiden, The Netherlands; 3 Department of Biostatistics, Erasmus Medical Center, Rotterdam, The Netherlands; University of California, Irvine, United States of America

## Abstract

The common approach to SNP genotyping is to use (model-based) clustering per individual SNP, on a set of arrays. Genotyping all SNPs on a single array is much more attractive, in terms of flexibility, stability and applicability, when developing new chips. A new semi-parametric method, named SCALA, is proposed. It is based on a mixture model using semi-parametric log-concave densities. Instead of using the raw data, the mixture is fitted on a two-dimensional histogram, thereby making computation time almost independent of the number of SNPs. Furthermore, the algorithm is effective in low-MAF situations.

Comparisons between SCALA and CRLMM on HapMap genotypes show very reliable calling of single arrays. Some heterozygous genotypes from HapMap are called homozygous by SCALA and to lesser extent by CRLMM too. Furthermore, HapMap's NoCalls (NN) could be genotyped by SCALA, mostly with high probability. The software is available as R scripts from the website www.math.leidenuniv.nl/~rrippe.

## Introduction

Genotyping algorithms for SNP chips can be partitioned roughly into two classes: 1) those that call genotypes for individual SNPs for a set of arrays and 2) those that call all SNPs for a single array. The first approach is the common one: for each SNP it collects pairs of fluorescence intensities for all arrays and applies a clustering algorithm. This is known as multi-array genotyping. In principle it has the advantage of being able to account for SNP-to-SNP variation. However, the number of available data points is limited to the number of samples: fewer data generally yield less reliable results. The latter problem is especially troubling if the SNP has a very low minor allele frequency (MAF), the minor allele being the one that has the lowest frequency in a given population. Low MAFs are known to have a detrimental effect on downstream analyses. Tabangin et al. [1] describe the latter for genome-wide association scans, but their results extend to other areas as well. Therefore, HapMap [2], [3] only targets MAFs of 5% and higher.

**Figure 1:**
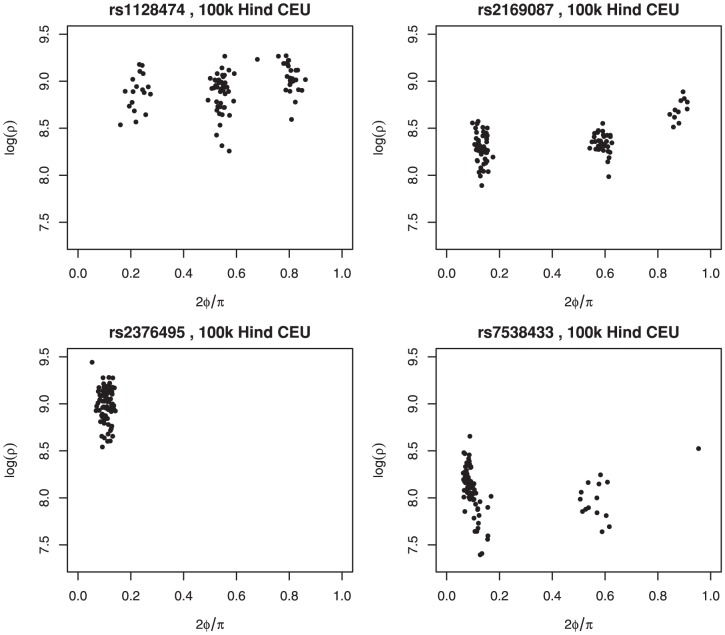
Multi-array genotyping for four separate SNPs in a sample Affymetrix 100 k Hind set from the CEU HapMap population. Top row: a clear three genotype division without minor allele frequency problem. Bottom row: genotype clusters with minor allele frequency problems.

In case of low MAF, there are very few or even no observations in some clusters. Figure 1 compares four SNPs. In the top row we clearly see three genotype clusters, which is not the case in the bottom row. There the panel at the left shows just a single cluster, while the third cluster in the right panel contains only one observation. A data transformation similar to that used in Illumina Beadstudio was applied. In this transformation the two signals for the two alleles are first transformed to polar coordinates (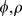) and displayed on modified scales: 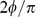 and 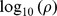. For the set of CEU samples on HapMap we find that respectively 13, 25 and 62% of the SNPs show 1, 2 or three genotypes,

It is clear that based on these 90 samples from the Central European (CEU) population, genotype calls for some SNPs can hardly be made effectively without the use of external information. For these reasons, common calling algorithms like BirdSeed [4] require 100 or more samples with known genotypes to train the model, while BRLMM-P and CRLMM [5], [6] require both a large number of samples as well as presence of all three genotypes AA, AB and BB. Still these methods have to accept a ‘No Call’ for some SNPs, due to high uncertainty.

A second approach is to cluster all SNPs on single arrays, using a mixture model. ALCHEMY [7] does *de novo* calling for small sets of samples. For each allele it introduces one-dimensional mixtures of normal distributions, one component for noise (when the allele is absent) and the other for the signal (when the allele is present). Wright et al. [7] work in the context of rice genotyping. They give an instructive overview of the problems connected with per-SNP genotyping, one of them being the absence of heterozygous genotypes, due to inbreeding.

Along similar lines, MAMS [8] combines multi-SNP and multi-array genotyping. A first step performs model-based clustering on all SNPs in a single array and a second step applies multi-array refinement of selected SNPs with unique hybridization properties (different from most SNPs). Mixtures of two-dimensional normal distributions are used. This is a time-consuming process, so sampling is used to get acceptable processing times. GenoSNP [9] is a mixture-based single-array genotyping algorithm for Illumina chips. All algorithms have in common that they use parametric models, i.e. normal distributions, for the mixture components. We propose a mixture of semi-parametric log-concave two-dimensional densities. We describe a fast algorithm and show its performance on HapMap data.

**Figure 2:**
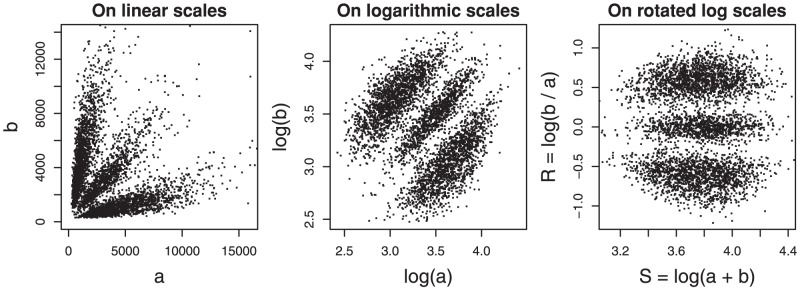
Illustration of signal transformation. Signal 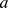 (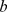) represents allele A (B). The left panel shows the signals on linear scales. The middle panel shows the same signals on logarithmic scale. The right panel shows transformed signals to 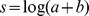 on the x-axis and 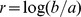 on the y-axis.

**Figure 3:**
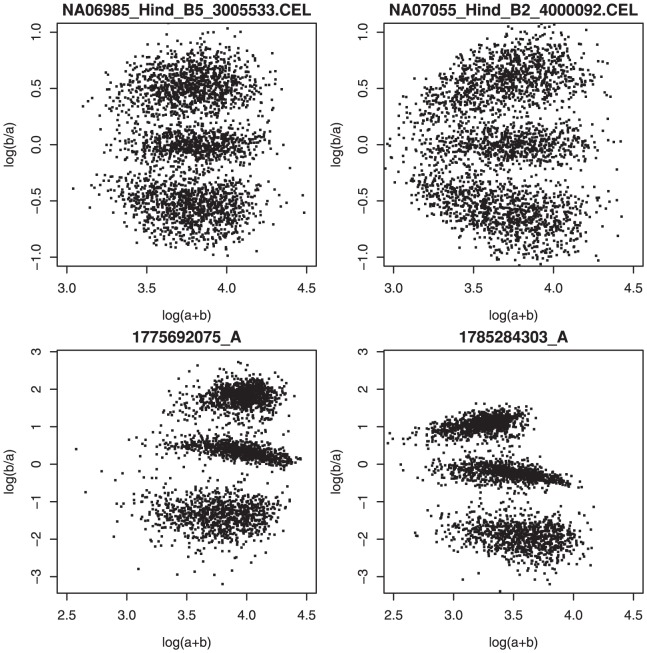
Single HapMap Affymetrix 100 k Hind samples (NA06985, NA07055 from left to right) in top panels, typical Illumina (HumanHap 550) arrays in bottom panels. SNPs are shown for chromosome 1.

We find it convenient to transform the allele channel signals to 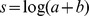 and 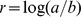 where 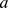 and 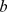 are fluorescence signals for allele A and B respectively (logs are to base 10). After this transformation (see Figure 2), three horizontal clusters are present, which correspond to the three possible genotypes. In Figure 3 results of the transformation are shown for two typical Affymetrix SNP6.0 (HapMap) arrays. The plots show a strong symmetry along the horizontal zero axis; this is to be expected, because Affymetrix uses a one-color fluorescence technology. In contrast, Illumina arrays, based on two different fluorescent dyes show a strong asymmetry as shown in Figure 3 for two typical Illumina HumanHap 550 arrays (source: department of Epidemiology, Erasmus Medical Center, Rotterdam, The Netherlands). For this reason we limit concentrate on Affymetrix arrays.

## Results

In this section we compare genotype calls from SCALA, GenoSNP and CRLMM with the consensus genotypes from HapMap. We explore call differences and evaluate SNPs that are not called by CRLMM and HapMap in terms of SCALA calls. We exclude the copy number probes. Our model has three clusters and so implicitly assumes normal DNA without copy number changes and null alleles.

We use probe set averages of the Affymetrix SNP6.0 CEL-files from the CEU population, CUPID set. To start the EM algorithm the data are split on the basis of 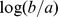. The splitting levels can be inferred visually from a few representative arrays and kept fixed. See the examples in Figure 2. We use −0.2 and 0.2, but these values are not very critical.

### Call agreements

**Figure 4:**
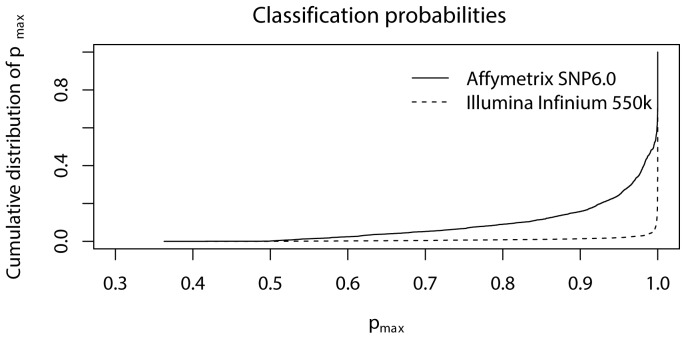
Cumulative distribution of the classification probability for typical Affymetrix and Illumina arrays. With a mixture of three components the minimum value that can be observed is 1/3.

Here we compare genotype calls from SCALA to those from HapMap. Our algorithm fits a mixture of three densities and computes for each SNP its probability of belonging to each of them. The largest of the three probabilities determines the assigned genotype. Figure 4 presents its cumulative distribution for typical arrays from Affymetrix and Illumina, showing that the classification probability is always large than 50%. It is possible to introduce a threshold and assign NN (NoCall) to the SNPs that score a maximum probability below it. However, we lack a principle for choosing a threshold, so we simply accept the classification. Note that, because all three probabilities are stored, any threshold can easily be applied later.

**Figure 5:**
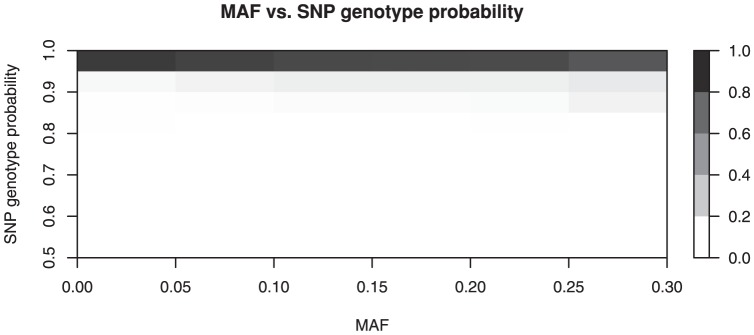
Two-dimensional histogram of 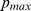 and MAF.

Figure 5 shows that 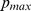 is not influenced strongly by the MAF. Here MAF is computed from the CEU sample of arrays.

Table 1 shows, as an example, the cross-table for chromosome 1 on array NA06985. SCALA and HapMap completely agree on the AA and BB genotypes. For the (HapMap) heterozygotes there is an 8.4% disagreement; this is 2.7% of the SNPs not classified as NN.

**Table 1:** Cross-tabulation of SCALA genotype calls (rows) and HapMap genotypes (columns) for chromosome 1 on array NA06989 (CUPID_p_HapMapPT06_GenomeWideSNP_6_A01_183598.CEL).

	AA	AB	BB	NN
AA	19029	633	0	97
AB	0	16820	0	139
BB	0	911	19326	110

**Figure 6:**
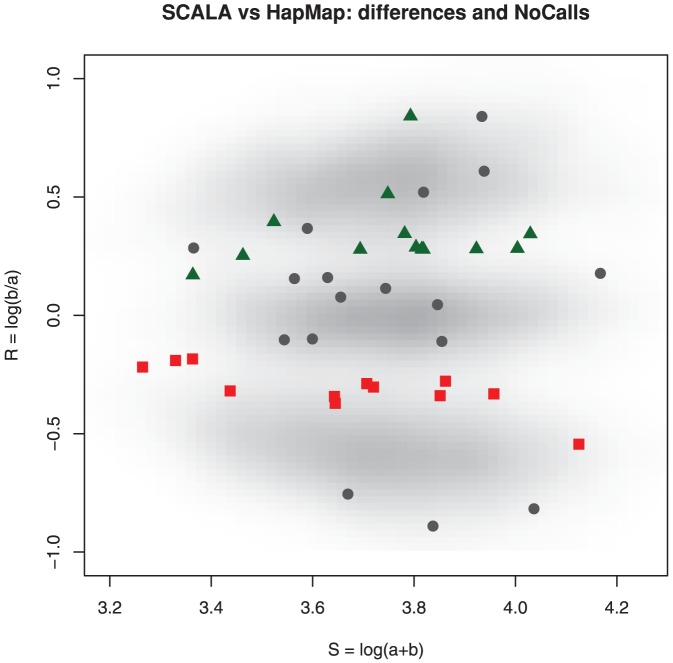
Example of SCALA call disagreements with HapMap for chromosome 1 on Affymetrix 100 k Hind array NA06991. Some Hapmap AB genotypes called as AA (red squares) or BB (green triangles) by SCALA. HapMap NN calls (circles) can be genotyped with high (open, 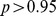) or low (filled, 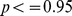) probability.

HapMap is the best we have to judge genotype calling algorithms, but it is not a gold standard. To put this in perspective, we study a small example, summarized in Table 2 and Figure 6. The data are for chromosome 1 on an Affymetrix 100 k Hind array (NA06991), and the number of disagreements is so small that we can clearly present the individual cases graphically.

**Table 2:** Cross-tabulation of SCALA genotype calls (rows) and HapMap genotypes (columns) for chromosome 1 in Affymetrix 100 k Hind: NA06991.

	AA	AB	BB	NN
AA	837	**12**	0	3
AB	0	731	0	9
BB	0	**13**	826	5

**Figure 7:**
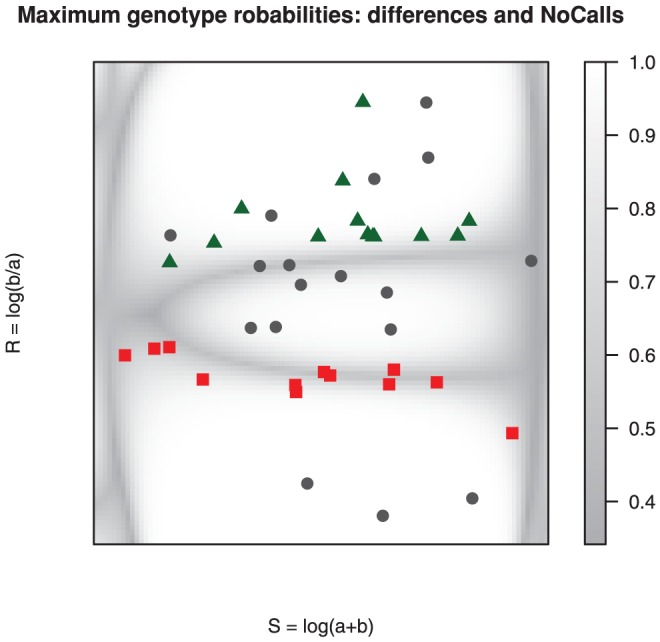
Example of SCALA call disagreements with HapMap for chromosome 1 on Affymetrix 100 k Hind array NA06991, now overlayed on the maximum of three cluster probabilities for each bin in the histogram. Hapmap AB genotypes called as AA (red squares) lie in a low maximum probability region of the array, while BB (green triangles) by SCALA do so only for a part. HapMap NN calls (circles) can be genotyped with high (open, 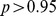) or low (filled, 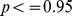) probability. The latter mostly lie in the low probability regions as well.

Figure 6 shows all SNPs as a gray density cloud [10], with the disagreements between SCALA and HapMap overlayed. After fitting the semi-parametric mixture we can compute and plot the maximum classification probability for each bin of the histogram. This is done in Figure 7, where the dark regions indicate low classification performance of SCALA. Again the disagreements are overlayed.

The message of Figure 7 seems to be the following. The BB genotype calls of SCALA all lie in the relatively high-probability region of the top cluster. We would accept these calls. The SCALA AA calls lie predominantly in the dark valley between the bottom and middle cluster. Essentially no verdict is possible here. The majority of HapMap NNs lie in high-probability regions, so it is surprising that they have not been called.

**Figure 8:**
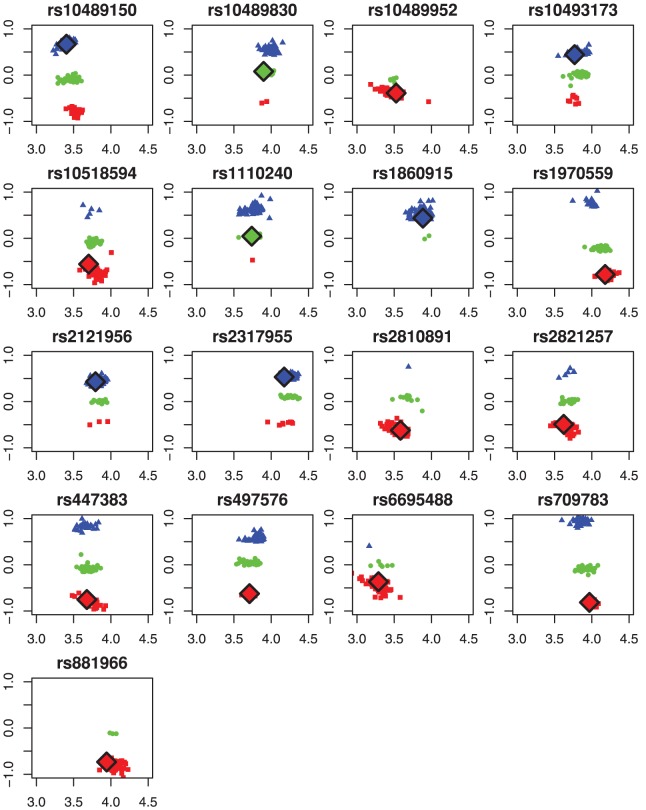
17 NN calls from HapMap as shown in Figure 5 plotted in single-SNP multi-array orientation. For at least 10 out of 17 we clearly see lack of one or more genotype clusters. NoCalls from HapMap for array NA06991 are overlayed with tilted squares and black borders. From both single- and multi-array genotyping point of view they seem to have a clear classification.

To understand this better, we have plotted in Figure 8 all arrays for each HapMap NN on array NA06991, using our choice of transformed fluorescence intensities. The symbols and colors represent the SCALA calls. Array NA06991 is represented by large diamonds with black borders. In the majority of cases one would expect a multi-array algorithm to work well, so it is surprising to see that no calls have been made by HapMap.

**Figure 9:**
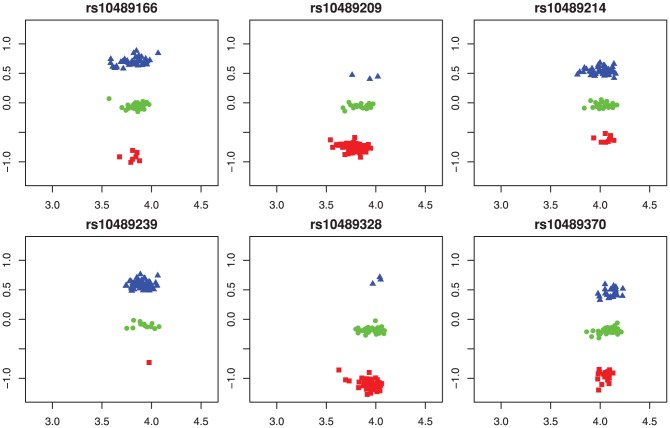
A selection of 6 SNPs with a high average classification probability, after single-chip genotyping, over all arrays.

**Figure 10:**
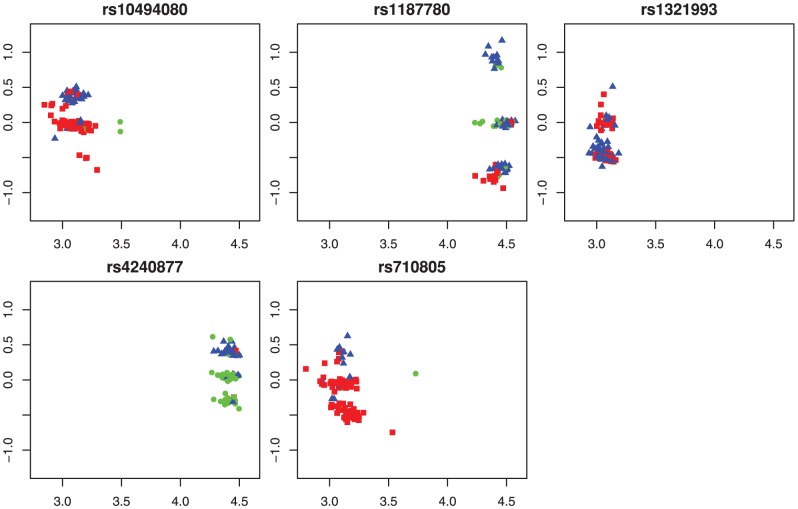
A selection of 6 SNPs with a low average classification probability, after single-chip genotyping, over all arrays.

Multi-array plots per SNP are useful, but unfortunately but their number is too large to handle. We present a selection from the two extremes of the spectrum from high to low quality. The average of 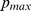 per SNP, over arrays, is a reasonable indicator of calling quality. Figure 9 shows the six best SNPs, with averages between 0.9902 and 0.994, while Figure 10 shows the six worst SNPs, with averages between 0.5187 and 0.5804. Based on these plots one would expect equally good results for the high-quality SNPs from multi-chip and single-chip algorithms. But a part of the low-quality SNPs we see three clusters and multi-chip genotyping looks feasible. The SCALA genotypes only partially agree with our visual impression of the clusters.

To provide a more general indication, we have calculated cross-tables as in Table 1 for all chromosomes on all 70 arrays in the SNP6.0 CUPID set for SCALA (Table 3) and for CRLMM (Table 4). Both tables are normalized to make column totals equal to 100%.

**Table 3:** Call agreement between SCALA (rows) and HapMap (columns), aggregated over all chromosomes in 70 arrays from the HapMap SNP6.0 CUPID set.

	AA	AB	BB	NN
AA	99.97	4.99	0.00	13.5
AB	0.03	90.11	0.00	69.1
BB	0.00	4.90	100.00	17.4

Numbers in percentages of HapMap genotypes; columns add up to 100%.

**Table 4:** Call agreement between CRLMM (rows) and HapMap (columns), aggregated over all chromosomes in 70 arrays from the HapMap SNP6.0 CUPID set.

	AA	AB	BB	NN
AA	100.00	2.85	0.00	19.2
AB	0.00	94.52	0.00	59.8
BB	0.00	2.59	100.00	21.0

Numbers in percentages of HapMap genotypes; columns add up to 100%.

We have also compared SCALA performance to the GenoSNP algorithm for Illumina arrays. The results on previously mentioned arrays from the Erasmus Medical Center, provided in Table 5, illustrate the power of the universal genotyping approach in SCALA. Equivalent performance is obtained using Illumina arrays from [11].

**Table 5:** Call agreement between SCALA (rows) and GenoSNP (columns), aggregated over all chromosomes in 20 arrays from the Erasmus Medical Center.

	AA	AB	BB	NN
AA	99.96	0.86	0.00	27.5
AB	0.04	98.52	0.02	49.1
BB	0.00	0.62	99.98	23.4

Numbers in percentages of GenoSNP genotypes; columns add up to 100%.

In summary we found that overall agreement between SCALA and HapMap is comparable to that of CRLMM. However, for the AB calls from HapMap we see differences in the direction of both AA and BB labels, for both SCALA and CRLMM, where the differences for SCALA were about twice as large, up to 4.99% of all HapMap ABs. However, after visual inspection of their location in their single array genotype clustering, for a large number of these differences it seems almost strange that they were called AB by HapMap: they lie in or close to the AA or BB cluster in the single array. In addition we found that for many genotypes that were not called in HapMap, probably due to problems with minor allele frequencies or low call probabilities we could call those SNPs with a probability larger than 0.95 in most cases. Further visual inspection revealed that those SNPs lie close to the center of one of the three clusters in a single array setting.

## Discussion

We presented a fast novel approach to call SNP genotypes in individual arrays using semi-parametric log-concave mixtures.

To assess performance we compared genotype calls from a multi-array method (CRLMM) and from our single-array method (SCALA) to a set of consensus genotypes from HapMap. The number of agreements and differences in terms of homo- and heterozygous calls showed that SCALA can be used to call genotypes efficiently and effectively. Even SNPs that were not genotyped in HapMap can be genotyped with reasonable certainty using a single chip. We also evaluated performance against the single-array algorithm GenoSNP, dedicated to Illumina chips. Strong agreement was found. We conclude that our approach can handle a variety of platforms and cluster shapes.

The semi-parametric densities in the model can adapt there shape easily and automatically to a variety of cluster shapes. We did not observe cases where our model did not work well. So we did have no need for a “catch-all” component like in GenoSNP, a uniform density to handle observations that fall out of the three main clusters. It would be little work to ad such a component to SCALA.

**Figure 11:**
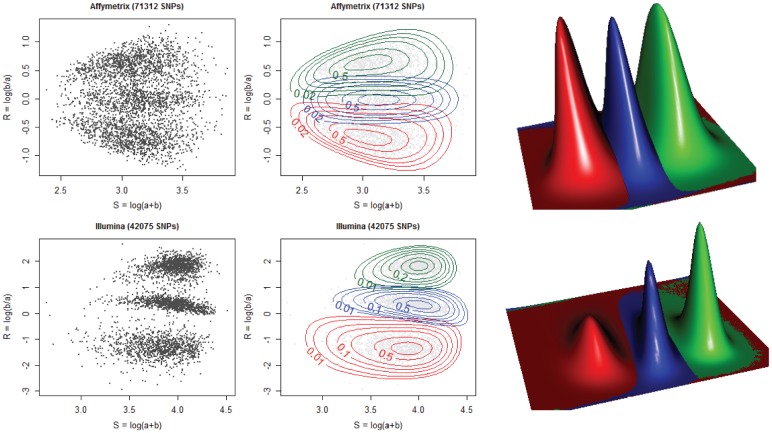
Top row: a typical Affymetrix SNP6.0 array. Bottom row: a typical IIlumina HumanHap550 array. Left panels : a random selection of 3500 SNPs on chromosome 1 plotted as dots. Middle panels: observations and contour lines of semi-parametric mixture components. Normalized contours (mode set to 1) are shown at [0.01, 0.02, 0.05, 0.1, 0.2, 0.5, 0.8]. Right panels: a 3D perspective of the smoothed densities.

The logistic advantages of singe-chip genotyping are large. Each array can be processed as soon as it becomes available; there is no need to wait until a large enough number has been collected. This is especially relevant when developing (new) chips for new or existing organisms. Quality control is also improved. It is easy to judge the estimated mixtures visually, using plots as in Figure 11.

## Methods

**Figure 12:**
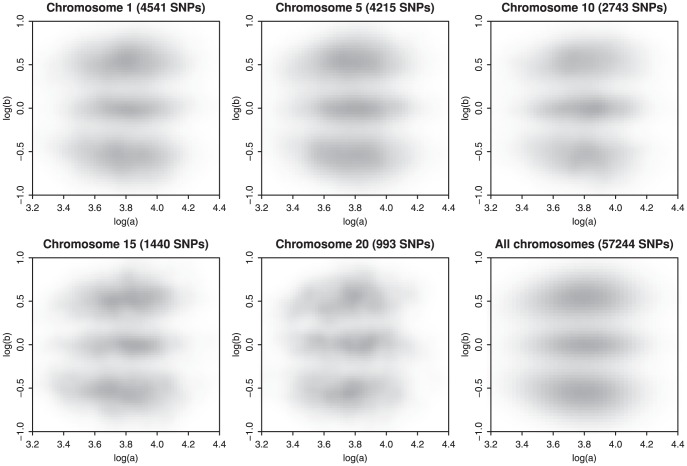
Genotype clusters in HapMap sample NA06985 (Affymetrix 100 k Hind enzyme only) for five individual chromosomes and genotype clusters over all chromosomes (bottom right panel). There is only a difference in SNP density, but not in scale or cluster separation.

In this section we describe how we fit a mixture of three two-dimensional semi-parametric log-concave densities to transformed fluorescence signals, as illustrated in Figure 3 [12]. In the case of an Affymetrix array the signals are summaries of probe sets, so we do not try to exploit any patterns in the signals from the individual probes. The reason is simple: we have no need for it. To avoid scatter plots showing three solid black clusters, we use data from one chromosome. This is only for illustrational purposes; it should be understood that all SNPs on one array are genotyped at the same time. Figure 12 illustrates the genotype cluster separation and their shapes for a selection of chromosomes as well the shapes for the complete array. As can be seen, they are very similar.

We describe in some detail how to fit a mixture of log-concave densities in one dimension, borrowing from [13]. Then we sketch the procedure in two dimensions.

To compute a smooth density for a one-dimensional data set, we first construct a histogram with many bins, say 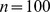. Let 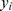 denote the count in bin 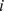 of the histogram and let 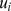 be the bin midpoint, with 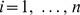. The vector of counts is denoted by 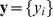. We write the expected count in bin 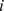 as 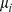, and assume that the counts have a Poisson distribution. To be sure that only positive expectations can occur, we work with 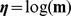. The vector 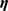 is constructed as a sum of B-splines:
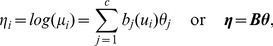
(1)where 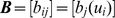 is an 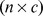 B-spline basis, with 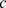, the number of bases, relatively large, say 20. Here, 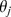 is the coefficient for each individual basis 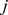.

Assuming a Poisson distribution for the counts, we maximize the penalized log-likelihood
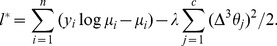
(2)The second term is a penalty on the third-order differences of the coefficients. The parameter 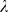 is used to tune smoothness. The larger 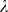, the stronger the influence of the penalty and the smoother the estimated density. This is the P-spline approach, advocated by [14], [15]. They also show that, with third-order differences in the penalty, 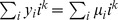, for 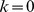, 1, and 2. This so-called conservation of moments means that, for all values of 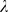, 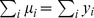, and that means and variance computed from 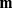 are equal to those computed from 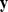. The latter property is very important, because it prevents the non-parametric density estimate 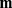 to deviate much from the observations. Most smoothers do not have this property; the variance of the estimated density increases with the smoothness. For components of mixtures this is an undesirable property.

Smoothness is tuned with the parameter 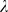. There are ways to optimize it in a data-driven way, using AIC, but in our application we trust our visual instinct. Here, we decide visually, because in practice we see that any reasonable amount of smoothing results in the same calls. The amount of smoothing mostly determines visual appeal. The third order differences also have the effect that for larger values of 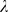 the vector 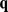 tends towards a quadratic series, because for such a series third order differences vanish and the penalty is zero. Unless the series of counts 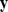 has a manifest J, U, or L shape, 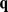 will approach a mountain parabola and the estimated density will show a unimodal log-concave shape. This is a desirable property for components of the mixtures we consider.

Setting the derivative of 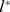 equal to zero gives
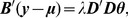
(3)where 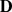 is a matrix of contrasts such that 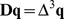. Linearization of (3) leads to
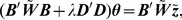
(4)where 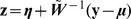 is the working variable, 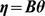, and 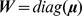; 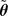, 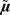 are approximations to the solution of (4). This system is iteratively solved until convergence, which usually is quick (less than ten iterations).

To estimate a mixture with three smooth components, we use the familiar EM (expectation-maximization) algorithm. Two steps are repeated until convergence: 1) split the counts 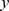 into three vectors of pseudo-counts, proportional to the current estimate of the mixture components; 2) apply smoothing to the pseudo-counts. A formal proof of convergence would require that we show that the penalized log-likelihood increases with each iteration. We did not try that. A hand-waving argument is that the M-step (fitting the semi-parametric component densities, using estimated weights) and the E-step (estimating the weights from the densities) are identical to the steps for fitting a discrete mixture of parametric densities, for which proofs of the convergence (to possibly a local maximum) of the EM method are available. In our experience we see that, given reasonable starting values, convergence is quick and reliable. It is easy to check results visually, showing contours of the densities and coloring dots according to their membership probabilities. Decent starting estimates for the components are needed. We will describe them for our application in what follows.

In two dimensions we use the same idea as described above, but now a two-dimensional histogram is formed, and the log of a density is formed by a sum of tensor products of B-splines. We sketch the adaptations that have to be made. Let 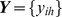 be an 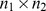 matrix of counts in a two-dimensional 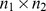 histogram. The center of bin 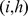 is given by (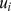, 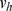). The expected values are modeled by sums of tensor product B-splines. Two bases are computed, 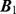, with 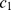 columns, based on 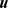 and 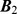, with 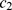 columns, based on 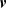. The bases are combined with a 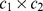 matrix 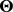 of coefficients, and the matrix of expected values is computed as
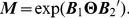
(5)Like in the one-dimensional case, a penalized Poisson log-likelihood is optimized. The penalty is more complex, because both rows and columns of 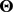 are penalized. If 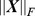 indicates the Frobenius norm of the matrix 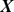, i.e. the sum of the squares of its elements, the penalty is
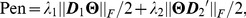
(6)where 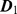 and 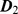 are matrices of the proper dimensions (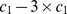 and 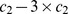) that form third differences.

One could vectorize 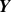, 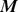 and 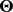 and form the Kronecker product of 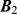 and 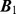 to mold the equations into a matrix-vector shape. It is, however, very inefficient to do this. Instead, we use the fast GLAM (generalized linear array model) algorithm [16], leading to enormous savings in time and memory use. The details are a bit involved, so we skip them here.

Our model is flexible enough to adapt to the quite different cluster shapes of different microarray platforms. Figure 11 shows results for an Affymetrix and for an Illumina array. Left panels show the raw observations, middle panels shows the density contours after estimation. The cluster contours represent the data well. The right panels show the smooth histograms in a 3D representation. Note how in the Illumina panel the density between the clusters is zero, while in the Affymetrix panel it is not. This can be seen in the genotyping probabilities as well, as discussed below.

The mixture components give three expected values for bin 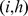 of the histogram: 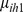, 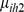 and 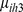. From these numbers follow, after division by their sum, three membership probabilities. The largest of the three, which we indicate by 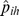 points to which cluster all the observations in the bin should be assigned. The distribution of 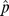 over all bins is a good indicator of classification confidence. Ideally all 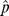 should be very close to one. Of course, strong confidence does not automatically mean good precision; that can only be assessed by comparison to a standard, as is done in the Results section.

Figure 4 shows the cumulative distributions of 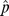 for the two arrays that we used as examples in Figure 11. Apparently the Illumina array generates more confidence. Keeping in mind the concentrated clusters in Figure 11 this is not a surprise.

The semi-parametric mixture model has a number of parameters that can be chosen by the user. For the histogram we advise a 100 by 100 grid. The domain of the histogram is covered by bases of 10+3 cubic B-splines (the additional three are for extra boundary splines). For the smoothing parameter we choose 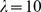. Our tests indicate that larger numbers of either bins or basis functions only increase computing time, but do not provide different calls. Furthermore, the algorithm is relatively insensitive to imperfect amounts of smoothing, as long as three more or less smooth densities are obtained.

To start the EM algorithm, we split the data in three groups by a very simple procedure. In the plot of 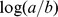 vs 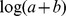 two horizontal lines are used to create three sectors (AA, AB and BB). This gives the pseudo-counts for the first round of density estimation. The positions of the separating lines are not very critical.

On a Core2 duo, level 2 cache 512 mb, 4 GB memory on a 64 bit OS, it takes around 20 seconds to call genotypes for a single Affymetrix SNP6.0 CEL file. This computation time includes building the histogram. Approximately the same time is needed for other arrays, almost independent of the number of SNPs, because the data are first summarized by a two-dimensional histogram. The latter is not a costly affair. We compared four strategies on one million pairs of uniformly distributed numbers (in parentheses times in seconds):

a loop over all observations (5.4);
a compiled version of the loop, using cmpfun (1.2);
using the function table() (5.6);
using a feature of the sparse matrix package package spam (0.31).

To put these numbers in perspective: the singular value decomposition of a 1000 by 1000 matrix takes 7.1 seconds on this computer. The memory footprint is small: it is far less than what is needed the one million pairs of fluorescence intensities for one chip.

Our genotyping algorithm has been implemented in R [17] as part of a larger software system, called SCALA.

## Supporting Information

Appendix S1 **We describe the translation step to match HapMap genotype calls to the SCALA {AA, AB, BB} format.** We compare genotype calls to those of Phase III. We only compare calls to SNPs that have matching ‘RSid's. almost half of the total. We disregard the four allelotypes (A,C,G,T) and refer to homozygous genotypes as AA or BB and the heterozygous as AB. Since genotype calls AA from either method are highly unlikely to be mistaken for BB, we can apply the above forced classification from the HapMap homozygous genotype calls into homozygous calls from SCALA.
(PDF)
