# Supplementary material for: Reliable Single Chip Genotyping with Semi-Parametric Log-Concave Mixtures
Source: PLoS One. 2012 Oct 16;7(10):e46267. doi: 10.1371/journal.pone.0046267 (PMC3473070; doi:10.1371/journal.pone.0046267)
Supplement: Appendix S1 — We describe the translation step to match HapMap genotype calls to the SCALA {AA, AB, BB} format. We compare genotype calls to those of Phase III. We only compare calls to SNPs that have matching ‘RSid's. almost half of the total. We disregard the four allelotypes (A,C,G,T) and refer to homozygous genotypes as AA or BB and the heterozygous as AB. Since genotype calls AA from either method are highly unlikely to be mistaken for BB, we can apply the above forced classification from the HapMap homozygous genotype calls into homozygous calls from SCALA. (PDF) [file pone.0046267.s001.pdf]

Appendix S1

Ralph C.A. Rippe[1], Paul H.C. Eilers[2] and Jacqueline J. Meulman[3]

We describe the translation step to match HapMap genotype calls to the SCALA {AA, AB, BB} format. We compare genotype calls to those of Phase III. We only compare calls to SNPs that have matching 'RSid's. almost half of the total. We disregard the four allelotypes (A,C,G,T) and refer to homozygous genotypes as AA or BB and the heterozygous as AB.

Since genotype calls AA from either method are highly unlikely to be mistaken for BB, we can apply the above forced classification from the HapMap homozygous genotype calls into homozygous calls from SCALA.

```
# create translation vector with default 5
# code contains the SCALA genotype calls
# rssel is a selection vector for matching SNP ids
# from HapMap SNP list, but in the SCALA ordering

# STEP 1:
  d = code[rssel]*0 + 5
# sort scala calls for available rs-ids in HapMap
# rsidt is the working list of HapMap rsids

# STEP 2:
  a = code[rssel][order(rsidt[rssel])]
# get aligned HapMap calls matched to rs-ids.
# hapmap is a dataframe with SNPs in rows,
# and arrays in colums
# hmsel is the SNP id list for the HapMap ordering

# STEP 3:
  b = hapmap[hmsel,samp+3][order(hapmap$rs[hmsel])]
# now a contains scala calls and
# contains hapmap calls for matching SNP id
# get all heterozygous calls

# STEP 4:
  selhetero = (b!='AA' & b!='CC' & b!='GG' & b!='TT')
# anything not homozygous is translated to 2 (AB)

# STEP 5:
  d[selhetero] = 2
# assign aligned homozygous calls


# STEP 6:
  d[a==1 & !selhetero] = 1
  d[a==3 & !selhetero] = 3
# keep NoCall seperate for later evaluation

# STEP 7:
  d[b=='NN'] = 4
```
